# The Effect of Boric Acid on Oxidative Stress, Inflammation, and Apoptosis in Embryonic and Fetal Tissues Damage Caused by Consumption of High-Fructose Corn Syrup in Pregnant Rats

**DOI:** 10.1007/s43032-025-01792-z

**Published:** 2025-01-16

**Authors:** Mehmet Başeğmez, Duygu Yüksel

**Affiliations:** 1 Department of Veterinary, Laboratory and Veterinary Health Program, Acıpayam Vocational High School, Pamukkale University, Denizli, Turkey; 2 Department of Medical Services and Techniques, Pathology Program, Vocational School of Health Services, Gümüşhane University, Gümüşhane, Turkey

**Keywords:** Boric acid, High fructose corn syrup, Inflammation, Oxidative stress, Rat

## Abstract

This study aimed to determine the protective role of boric acid in a pregnant rat model of high fructose corn syrup consumption. Consumption of high fructose corn syrup has been associated with adverse health outcomes in humans and animals. Twenty-eight healthy female Wistar albino rats (250–300 g weight and 16–24 weeks old) were randomly distributed into four equal groups (*n* = 7): Control, Boric acid (BA), High Fructose Corn Syrup (HFCS), HFCS + BA. Boric acid (20 mg/kg) was administered to pregnant rats via oral gavage every day during pregnancy. The prepared 30% HFCS (F30) solution (24% fructose, 28% dextrose) was added to the drinking water throughout pregnancy. At the end of pregnancy (day 19), blood, placenta, uterus, and fetuses were collected from rats. The results indicated that HFCS increases oxidative stress by increasing the level of MDA and decreasing GSH, SOD, and CAT activity in the blood of maternal. However, BA administration significantly decreased MDA levels and increased GSH levels, SOD, and CAT activity (*p* < 0.05). In addition, HFCS consumption significantly increased plasma TNF-α, IL-6, and leptin levels compared to control, BA, and HFCS + BA groups (*p* < 0.05). However, BA administration significantly decreased plasma TNF-α, IL-6, and leptin levels (*p* < 0.05). Furthermore, BA (20 mg/kg) significantly decreased HFCS-induced histopathological and immunohistochemical alterations in the placenta, uterus, and fetal tissue. In conclusion, BA may prevent HFCS toxicity in maternal and fetal tissues, as it regulates oxidative imbalance in pregnant rat and alleviates histopathological and immunohistochemical changes. The findings indicate a need for further studies to assess the potential of boron in preventing or mitigating the effects of HFCS during pregnancy.

**Graphical Abstract:**

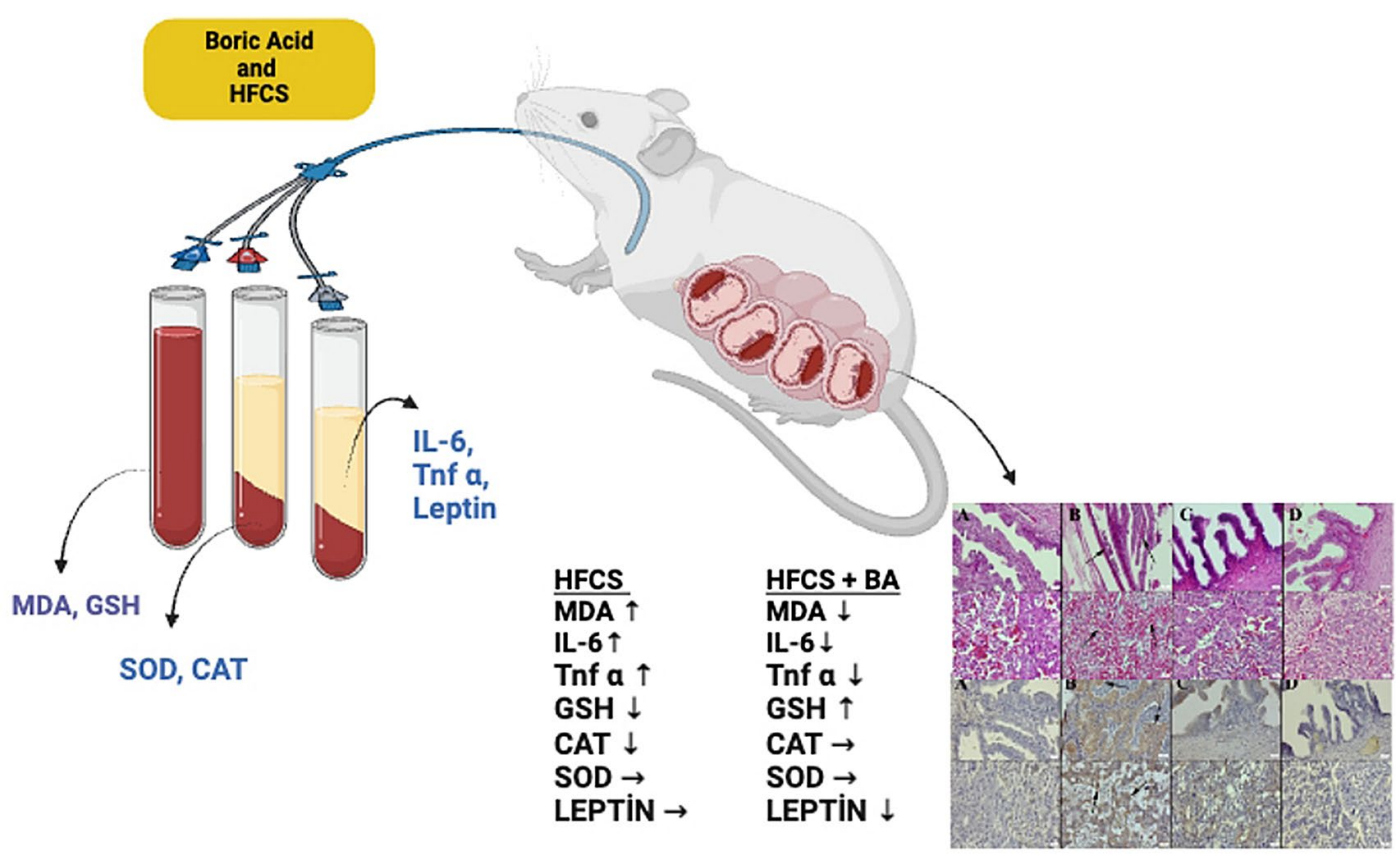

**Supplementary Information:**

The online version contains supplementary material available at 10.1007/s43032-025-01792-z.

## Introduction

Fructose, which can be found in fruits and some vegetables, is now widely used as a sweetener in the food and pharmaceutical industries. High fructose corn syrup (HFCS) is produced by hydrolyzing corn starch with glucoamylase and α-amylase, and partially isomerizing glucose to fructose [1]. HFCS is used as an industrial sugar to sweeten processed foods and beverages, especially dairy desserts, ice cream, snack foods, frozen dinners, soft drinks, and fruit juice [2]. The use of HFCS in processed, packaged, and unpackaged food and beverage products has been increasing since 1960 due to its ease of supply, stability, taste, and color properties [3]. HFCS, a commonly used sweetener, is believed to have significant detrimental effects on human health [4]. Researchers have shown that using HFCS during pregnancy causes insulin resistance and hyperlipidemia in the offspring [5]. Sweetened beverages containing significant levels of HFCS have been associated with adverse health outcomes, leading to excessive weight gain in humans and animals [6]. Additionally, Zargaraan et al. [2] stated in their statement that high HFCS consumption has been associated with increased obesity rates due to the breakdown of fructose in the organism faster and differently than glucose. Consumption of carbonated drinks with high fructose content during pregnancy has been linked to an increased risk of metabolic disease and obesity in the offspring [7, 8]. Mukai et al. [9] demonstrated that fructose intake during gestation elevates the expression of regulatory element binding protein-1c, which governs cholesterol and fatty acid synthesis in the liver of progeny, resulting in dysregulated lipid metabolism in later life. On the other hand, female offspring of mice consuming fructose from the beginning of pregnancy developed obesity, fatty liver, high serum leptin, and low serum adiponectin levels, while male offspring developed hypertension and insulin resistance [10]. Reports also indicate that female fetuses exposed to a high-fructose diet by the mother experience placental weight losses [11]. Therefore, high fructose intake during pregnancy has clear adverse effects on the fetus during pregnancy and on the health of the child after pregnancy.

Boron is abundant in nature and is considered an essential element in living organisms [12]. Boron compounds are most commonly available in commercial forms as boric acid (BA), disodium tetraborate (borax), colemanite, urexite, and sodium perborate [13]. Boron and its compounds have been demonstrated to be beneficial for both humans and animals in a variety of experimental investigations [14, 15]. Khaliq et al. [16] demonstrated the effect of boron on reprogramming cell metabolism and its potential to regulate inflammation and the immune system. Boron is also an important element for bone health due to its interactions with calcium, phosphor, vitamin D, and magnesium [17, 18]. Other studies have shown that boron regulates the hormone system and other biochemical markers, Kucukkurt et al. [19], in addition to reducing oxidative stress and DNA damage [20]. Additionally, studies have shown that whereas the fetuses of animals fed a boron-free diet develop slowly, those of animals treated with boron do so more rapidly and significantly [13].

This study aims to investigate the impact of an HFCS diet consumed from the onset of pregnancy on maternal uterus and offspring, as well as the therapeutic effects of boron, through histopathological and biochemical methods. Additionally, it examines how BA and HFCS influence embryonic tissue development, trophoblast cell proliferation, inflammation, and oxidant-antioxidant markers.

## Materials and methods

### Materials

#### Chemical

Boric acid (H_3_BO_3_) (Code number: V55901) was purchased from Chemistry Lab Istanbul, Turkey. The F30 form of HFCS, including 24% fructose and 28% dextrose, was acquired from Toposmanoğlu (Isparta, Turkey). During the study, the cage housing the HFCS and HFCS + BA groups received daily additions of F-30 solution (30% fructose) to their drinking water.

## Experimental Protocol

### Animals and Experimental Model

Twenty-eight Wistar-Albino female rats (250–300 mg, 16–24 weeks old) were gotten from the Pamukkale University Experimental Surgery Application and Research Center. They were kept in a room with a temperature of 22 ± 1 °C, a humidity of 50 ± 5%, a 12-hour light-dark cycle, and a standard pelleted rodent diet. Diet and drinking water for rats were given ad libitum. The experiment adhered to the ARRIVE 2.0 guidelines. Pamukkale University’s ethics committee approved this study (protocol number PAUHDEK-2023/16). The rats were checked daily at regular intervals under the supervision of a veterinarian.

Rats were divided into four experimental groups, each containing seven rats (*n* = 7); control, boric acid (BA), high fructose corn syrup (HFCS), and HFCS + BA. After a week of adaptation, female rats mated with male rats. The vaginal smears of the animals were analyzed for sperm presence, and the smear identified as sperm-positive was designated as the first day of pregnancy. Then, HFCS, which was freshly prepared every day during pregnancy, was added to the drinking water at the dose specified by Topsakal et al. [21] and shown in Table 1. Boric acid was administered orally at a dose of 20 mg/kg throughout pregnancy [14]. The control group was administered water by oral gavage. Table 1 presents the experimental process and the experimental groups.

**Table 1:** The procedure process and experimental groups

Groups	1st-19th days	19th day	
Control	1 ml of water was administered via gastric gavage until the 19th day of pregnancy.	At the end of pregnancy (day 19), rats were sacrificed.	
Boric acid (BA)	20 mg/kg boric acid was administered by gastric gavage until the nineteenth day of pregnancy.		
High Fructose Corn Syrup (HFCS)	High fructose corn syrup (30%) was administered until the nineteenth day of pregnancy.		
HFCS + BA	High fructose corn syrup (30%) and boric acid (20 mg/kg) were administered until the nineteenth day of pregnancy.		

At the end of the study, pregnant rats were euthanized under anesthesia with ketamine (87 mg/kg) and xylazine (13 mg/kg), and blood samples were collected. Plasma was obtained from blood samples taken for biochemical analysis and stored in the freezer at -80 °C until analysis. The uterus, placenta, and fetal tissues (brain, liver, kidney, and lungs) of the euthanized pregnant rats were collected, and tissue samples were fixed in 10% formalin for histopathological analysis. The collected tissue samples were analyzed histopathological and immunohistochemically.

### Preparation of Erythrocytes

The erythrocytes were precipitated within 30 min of blood collection by centrifugation at 3500 rpm for 15 min at 4 °C, and the plasma was separated. Erythrocytes were washed three times with isotonic serum and the raised layer was removed. Then, the equal volume of isotonic serum and erythrocytes was placed in Eppendorf tubes and stored in a deep freezer at -80 °C until analysis [22]. Malondialdehyde (MDA) and glutathione (GSH) levels were measured in whole blood. Superoxide dismutase (SOD) and catalase (CAT) activities were measured using erythrocyte homogenate samples.

## Methods

### Biochemical Analysis

Lipid peroxidation was assayed by measuring the level of MDA in whole blood. MDA levels was determined using the method described by Draper and Hadley and expressed as nmol/ml [23]. The GSH content of whole blood was measured using the Beutler et al. method, and the results were expressed as nmol/mL [24]. The SOD activity was determined utilizing the method established by Sun et al. [25], expressed as U/mgHb. The CAT activity was determined using Luck’s method [26], and it was defined as U/mgHb.

### Determination of TNF-α, IL-6, and Leptin

Plasma TNF-α (Cat. No. E0764Ra), IL-6 (Cat. No. E0135Ra), and leptin (Cat. No. E0561Ra) levels were determined using a commercial rat ELISA kit from Bioassay Technology Laboratory, according to the manufacturer’s instructions. Following the color change in the wells, the degree of enzymatic degradation of the substrate was assessed using absorbance measurement at 450 nm.

### Histopathological Analysis

During necropsy, uterus, placenta, and fetal samples were obtained, evaluated macroscopically for any abnormal features, and then fixed in a 10% neutral formalin solution. The samples were processed through routine tissue tracking procedures in a tissue processor (Leica, Wetzlar, Germany), then embedded in paraffin to obtain paraffin blocks. After cooling, 5 μm-thick portions of the paraffin blocks were cut out using a Leica RM2155 rotary microtome. Hematoxylin-eosin (HE) staining was used to analyze these sections under a light microscope.

A semiquantitative grading system that graded hyperemia, hemorrhage, inflammatory cell infiltrations, degenerative changes, and necrotic alterations was used to assess the histological lesions in the tissues. Mild, moderate, and severe affections were given values ranging from 0 to 3, and normal tissues were graded as normal. Table 2 shows the scoring standards for histopathologic changes.

**Table 2:** Histopathological grading scores

Score	Description
0	No lesion
1	Mild hyperemia, slight hemorrhage, no inflammation, and vacuolar degeneration
2	Severe hyperemia, slight hemorrhage, slight inflammation, and slight necrosis
3	Severe hyperemia, severe hemorrhage, marked inflammation, and marked necrosis

### Immunohistochemical Examination

One set of sections was taken from each block and placed on poly-L-lysine-coated slides before being stained for caspase-3 expression using the streptavidin-biotin method per the manufacturer’s instructions using an anti-caspase-3 antibody (EPR18297; Abcam, Cambridge, UK). Using biotinylated secondary antibodies and streptavidin-alkaline phosphatase conjugate, immunohistochemistry was performed on the sections after the primary antibodies were diluted to 1/100 and treated with them for 60 min. The secondary antibody used was the EXPOSE Mouse and Rabbit Specific HRP/DAB Detection IHC Kit (ab80436) from Abcam in Cambridge, UK. The chromogen used was diaminobenzidine (DAB). Instead of using primary antibodies, an antigen dilution solution was used as a negative control. The pathological analyses were independently evaluated by a pathologist from another university through a blind review process. The percentage of cells that were positively immunostained for caspase-3 in 10 distinct fields for each slice for all groups was calculated at an objective magnification of X40. Counting was done on the image analyzer’s output using the ImageJ program (National Institutes of Health, Bethesda, MD, version 1.48). The photos were separated into color channels, cropped, and any artifacts eliminated before counting. Cells within the regions of interest were counted using the software’s counting tool after being picked using a selection tool. Only cells that displayed intense brown staining were regarded as positive, and the brown hue was employed to determine positive staining. Microphotographs were taken using the Database Manual Cell Sens Life Science Imaging Software System (Olympus Co., Tokyo, Japan).

### Statistical Analysis

Statistical analysis was performed using the SPSS 27.0 (IBM SPSS Statistics, IL, USA) statistical package. Data are shown as mean ± standard error mean (SEM), 95% confidence interval or percentage. GraphPad Prism 10.1.1 (GraphPad Software, San Diego, CA, USA) was used for graphical presentations. One-way analysis of variance (ANOVA) was used to compare independent multiple groups, and the level of significance between groups was determined by the Duncan post hoc test. A p-value of less than 0.05 was considered “significant,” whereas a p-value of more than 0.05 was considered “not significant.”

## Results

### Body Weight

The body weight changes of pregnant rats receiving HFCS and BA supplementation are shown in Table 3. In Table 3, it was observed that the body weight gain of rats in the control, HFCS, and HFCS + BA groups increased periodically during pregnancy, and the body weight gain in the 3rd period of pregnancy increased significantly compared to the 1st period (*p* < 0.05). Boric acid had no significant effect on body weight levels in pregnant rats (*p* > 0.05).

**Table 3:** The effect of BA and HFCS on body weight levels in pregnant rats (*n* = 7, mean ± SEM)

Period (days)	Control	BA	HFCS	HFCS + BA
(1. Period) 1–6	248.43 ± 6.19^b^	281.14 ± 4.68	253.29 ± 10.29^b^	260.43 ± 8.76^b^
(2. Period) 6–12	266.86 ± 7.71^ab^	295.00 ± 5.12	268.57 ± 10.98^ab^	276.42 ± 8.79^ab^
(3. Period) 12–18	281.00 ± 10.45^a^	289.14 ± 4.51	286.57 ± 7.91^a^	297.86 ± 9.80^a^

The different superscripts a and b showed statistically significant differences within the same column (*P* < 0.05). The same superscripts a and b did not show statistically significant differences within the same column (*p* > 0.05). The values were expressed as means ± SEM. BA: Boric acid; HFCS: High Fructose Corn Syrup

### Effects of BA and HFCS on MDA and GSH Levels

**Fig. 1:**
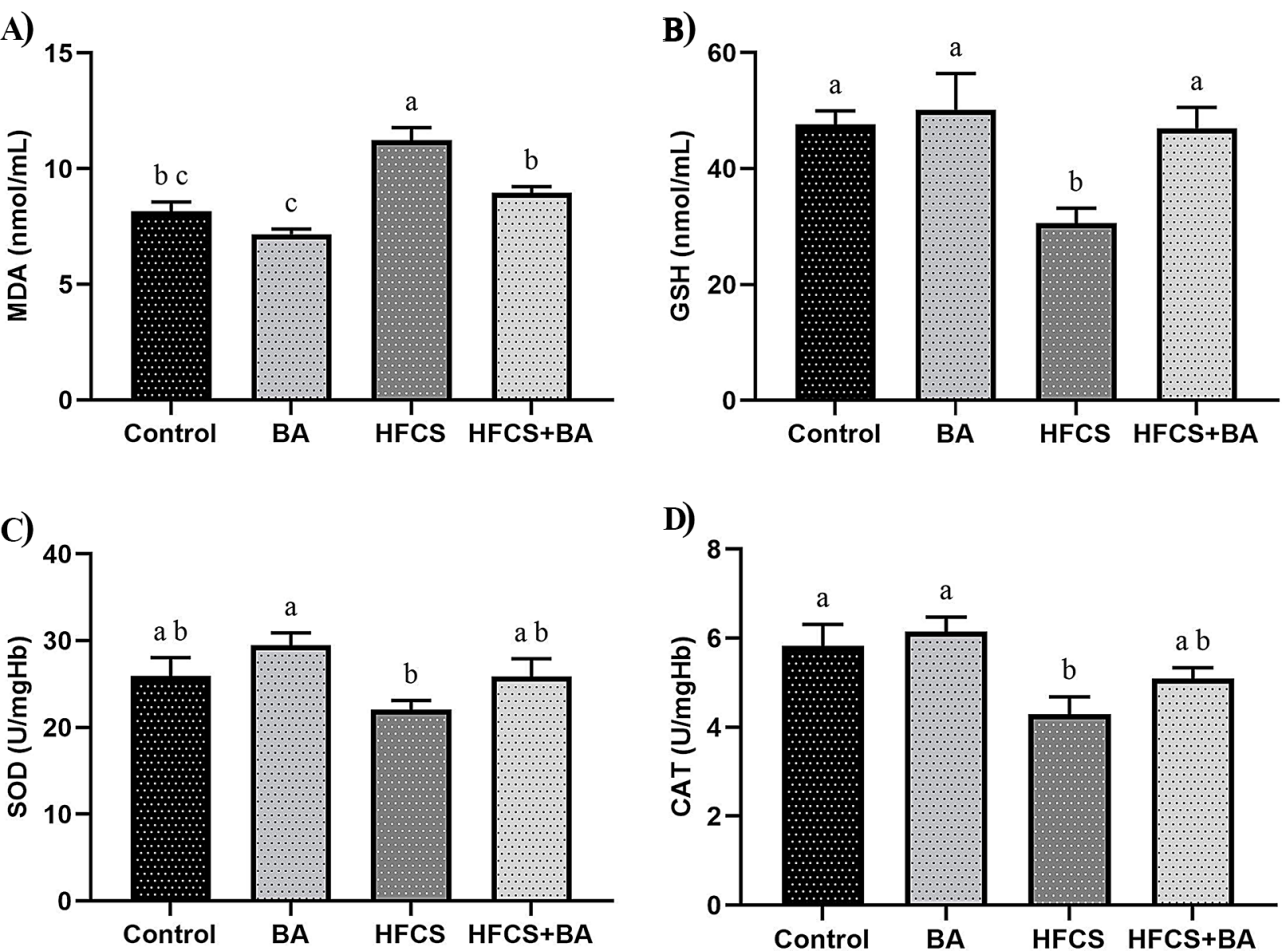
Effects of BA on whole blood MDA (**A**), GSH (**B**) levels and erythrocyte homogenate SOD (**C**), CAT (**D**) enzyme activities in HFCS-induced pregnant rats. **BA**: Boric acid; **HFCS**: High Fructose Corn Syrup; **MDA**: Malondialdehyde; **GSH**: Glutathione; **SOD**: Superoxide Dismutase; **CAT**: Catalase. ^**a, b, c**^: Different letters in the same column were statistically significant, *p* < 0.05

In the whole blood (Fig. 1), HFCS significantly increased MDA levels compared to the control and BA groups (*p* < 0.05). BA significantly decreased MDA levels in the HFCS + BA group (*p* < 0.05). HFCS significantly decreased GSH levels compared to the control and BA groups (*p* < 0.05). BA significantly increased GSH levels in the HFCS + BA group (*p* < 0.05).

### Effects of BA and HFCS on SOD and CAT Enzyme Activity

HFCS had no significant effect on SOD enzyme activity compared to the control group (*p* > 0.05). BA increased SOD enzyme activity in the HFCS + BA group, but the differences were not statistically significant (*p* > 0.05). HFCS significantly reduced CAT levels in the HFCS group compared to the control and BA groups (*p* < 0.05). BA increased CAT levels in HFCS + BA groups. However, it was not statistically significant (*p* > 0.05).

### Evaluation of TNF-α, IL-6, and Leptin Levels

**Fig. 2:**
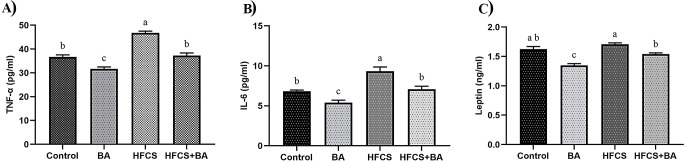
Effects of BA on plasma TNF-α (**A**), IL-6 (**B**), and leptin (**C**) levels in HFCS-induced pregnant rats. **BA**: Boric acid; **HFCS**: High Fructose Corn Syrup; **TNF-α**: Tumor Necrosis Factor Alpha; **IL-6**: Interleukin-6. ^**a, b, c**^: Different letters in the same column were statistically significant, *p* < 0.05

As shown in Fig. 2, HFCS caused a significant increase in TNF-α levels compared to the control group (*p* < 0.05). BA reduced TNF-α levels to statistical significance in both the BA group and the HFCS + BA group (*p* < 0.05). As illustrated in Fig. 2, HFCS caused a significant increase in IL-6 levels compared to the control group (*p* < 0.05). BA significantly decrease IL-6 levels in the BA group and the HFCS + BA group (*p* < 0.05). As demonstrated in Fig. 2, plasma leptin levels increased in the HFCS group compared to the control group, but the difference was not statistically significant (*p* > 0.05). BA significantly decreased leptin levels in the BA group and the HFCS + BA group (*p* < 0.05).

### Histopathological Examination

**Fig. 3:**
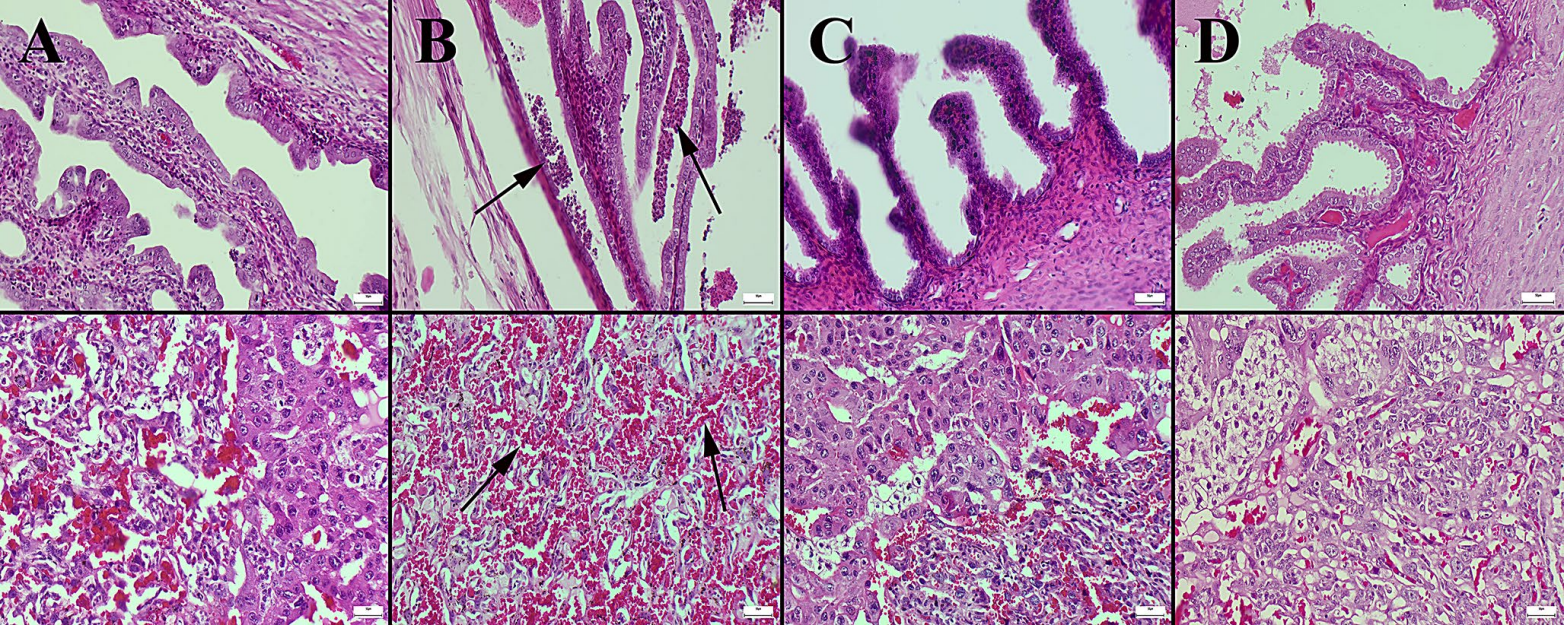
Histopathological appearance of the uterus (upper row) and placental tissues (below row) between the groups. (**A**) Normal tissue histology in the control group. (**B**) Marked inflammatory cell infiltrations (arrows) in uterus the and severe hemorrhage in placental tissue (arrows) in the HFCS group. (**C**) Decreased pathological findings in HFCS + BA group. (**D**) No lesions in BA group, HE, Scale Bars = 50 μm. BA: Boric acid; HFCS: High Fructose Corn Syrup

**Fig. 4:**
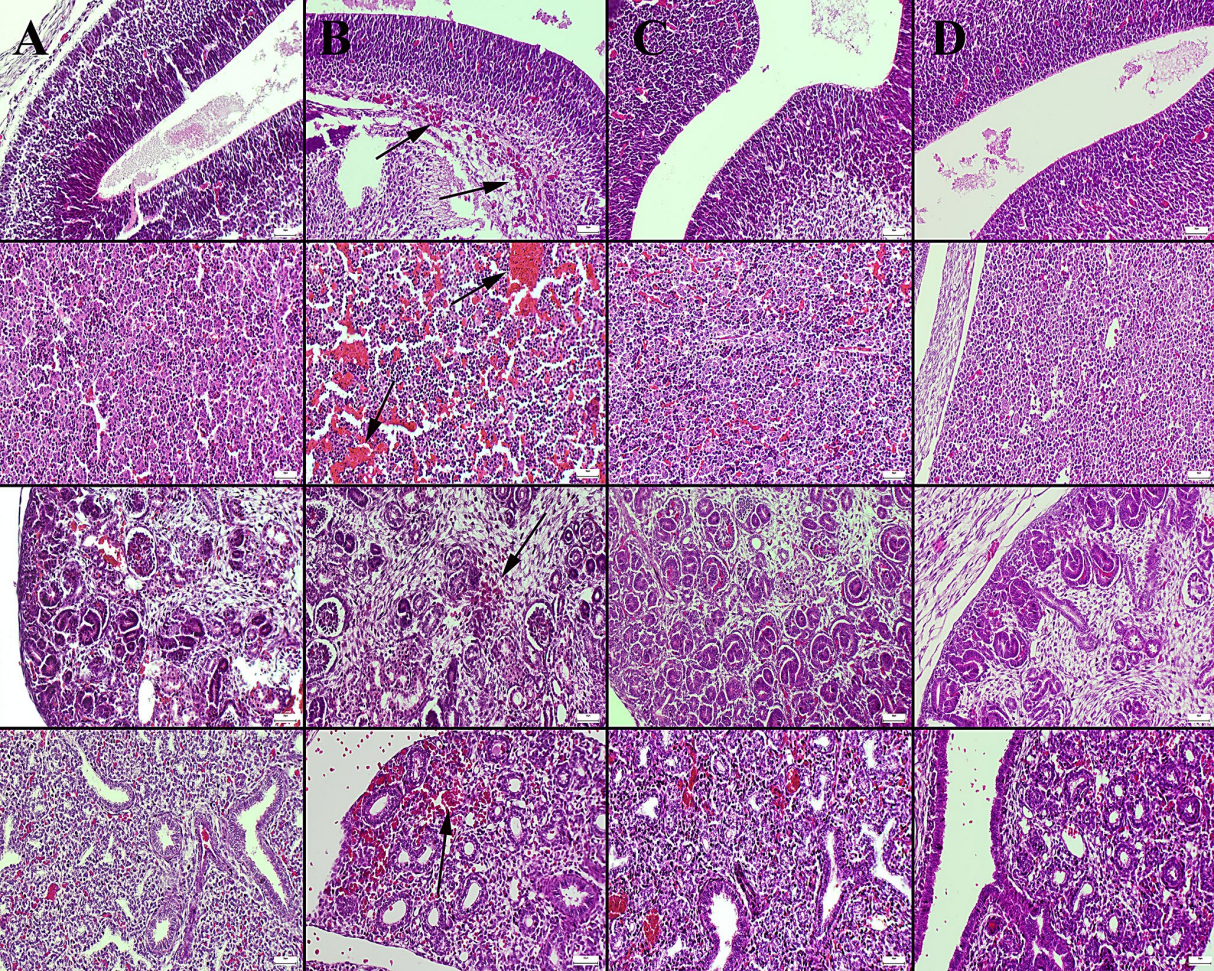
Histopathological appearance of the fetal tissues (brain cortex first row; liver second row; kidney third row and lungs fourth row) between the groups. (**A**) Normal tissue histology in the control group. (**B**) Marked hyperemia and hemorrhage (arrows) in the HFCS group. (**C**) Decreased pathological findings in the HFCS + BA group. (**D**) No lesions in BA group, HE, Scale Bars = 50 μm. BA: Boric acid; HFCS: High Fructose Corn Syrup

The histopathologic changes in the maternal uterus and placental tissues of the rats in the experimental groups were described in detail and shown in Fig. 3, respectively. Histopathologic changes in fetal brain, liver, kidney and lung tissues are described in detail and shown in Fig. 4. During histological analysis, normal maternal and fetal tissue histology was seen in the control and BA groups (Figs. 3 and 4). The maternal and fetal tissues of the HFCS group, however, showed substantial hyperemia, microhemorrhages, and mild to severe inflammatory cell infiltration (Figs. 3 and 4). Boric acid significantly reduced histopathological damage in maternal and fetal tissues in the HFCS + BA group (Figs. 3 and 4). Table 4 displays the results of the statistical analysis of histopathological findings.

### Immunohistochemical Analyses

**Fig. 5:**
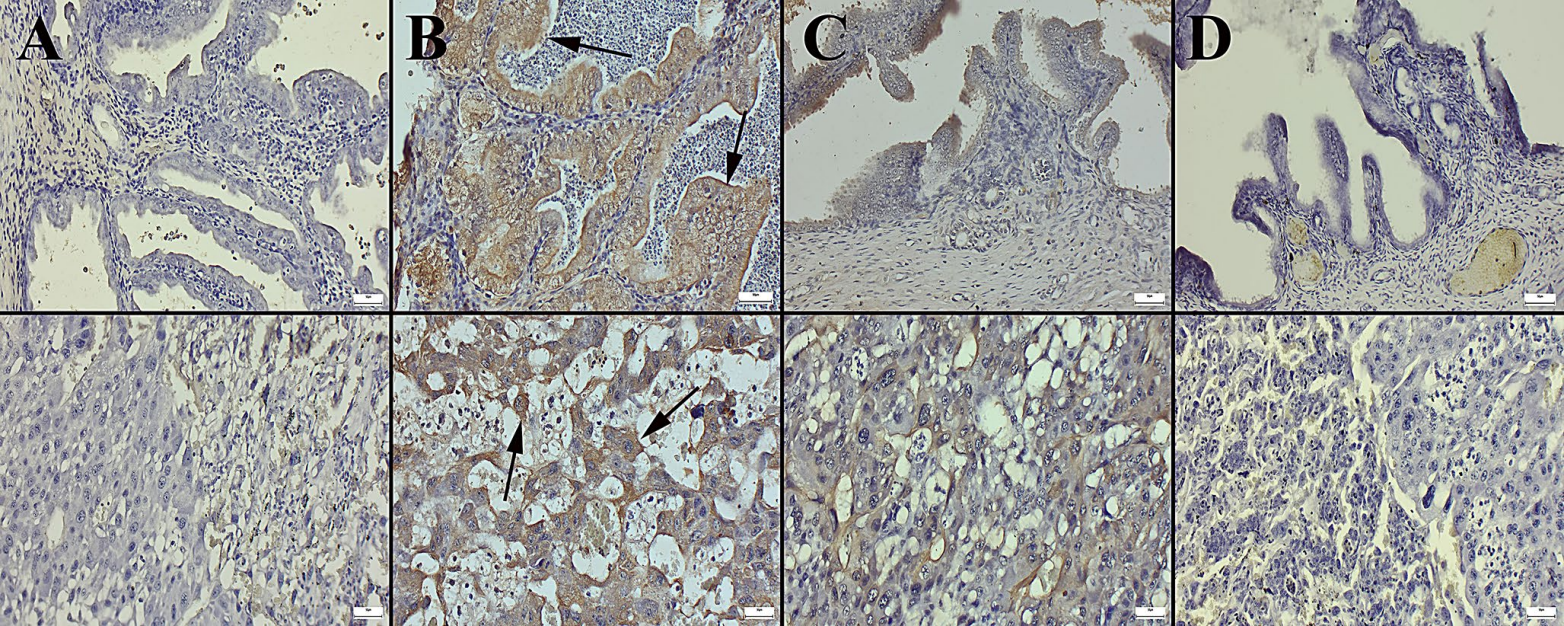
Representative caspase-3 immunohistochemical findings in the uterus (upper row) and placental tissues (below row) among the groups. (**A**) Negative expressions in the control group. (**B**) Marked increase in expressions (arrows) in the HFCS group. (**C**) Almost negative expressions in the HFCS + BA group. (**D**) No expression in the BA group, Streptavidin Biotin Peroxidase Method, Scale Bars = 50 μm. BA: Boric acid; HFCS: High Fructose Corn Syrup

The microscopical examination of caspase-3 immunostained uterus and placental slides revealed no immunopositive cells in the control and BA groups, while a marked increase in expression was observed in the HFCS group. However, BA treatment significantly reduced the expression in the HFCS + BA group (Fig. 5).

**Fig. 6:**
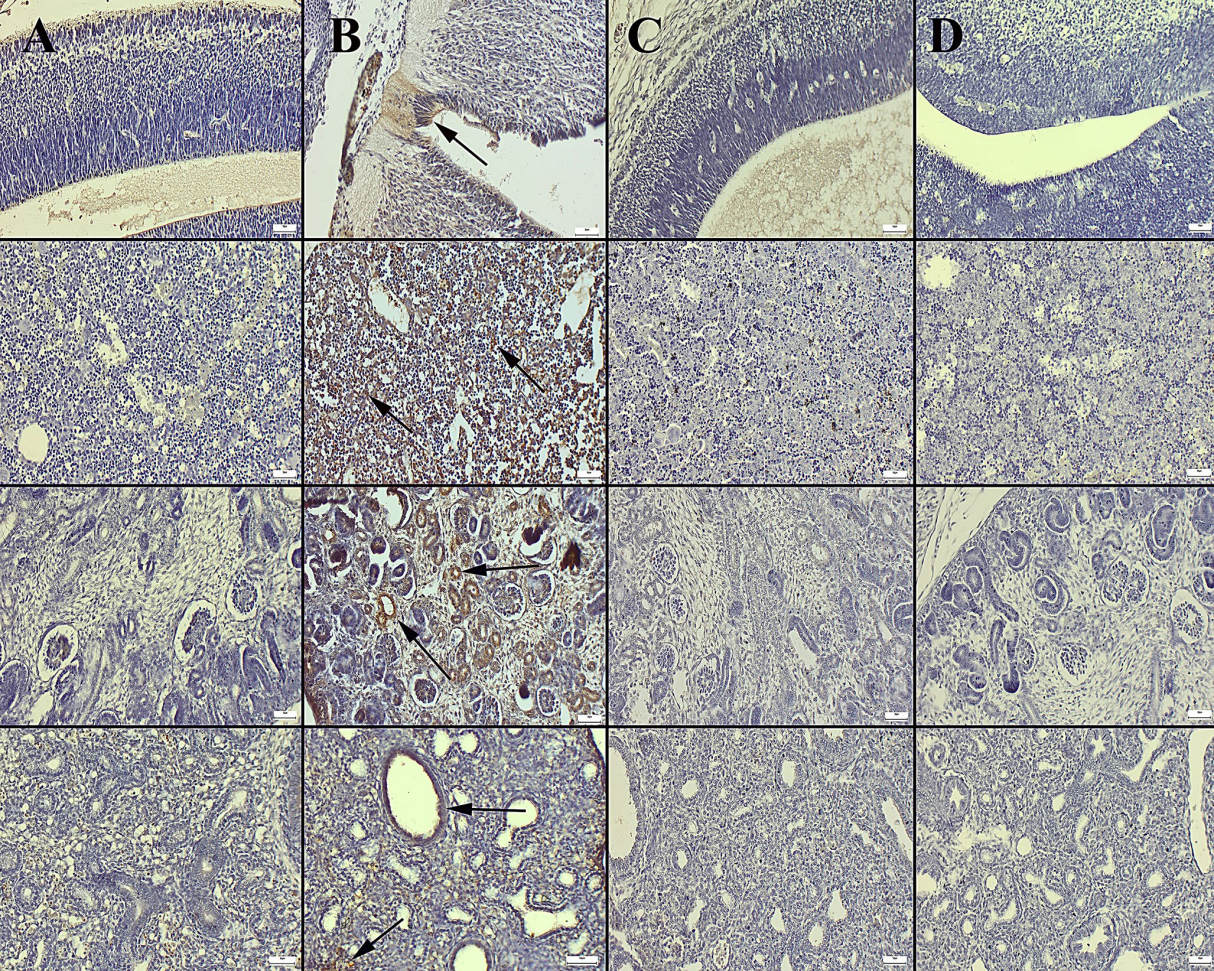
Cas-3 immunohistochemistry results in the fetal tissues (brain cortex, first row; liver, second row; kidney, third row and lungs, fourth row) between the groups. (**A**) No expressions in the control group. (**B**) Severe increase in expressions (arrows) in the HFCS group. (**C**) Decreased expressions in the HFCS + BA group. (**D**) Negative expression in the BA group, Streptavidin Biotin Peroxidase Method, Scale Bars = 50 μm. BA: Boric acid; HFCS: High Fructose Corn Syrup

Upon microscopical examination of caspase-3 immune-stained fetal tissues (brain cortex, liver, kidney and lungs) no or very slight number immune positive cells were observed in the control and BA groups. However, a marked increase in expression was noted in the HFCS group. Notably, BA treatment significantly reduced the expressions in the HFCS + BA group (Fig. 6).

**Table 4:** Statistical analysis of histopathologic and immunohistochemical scores between groups

	Tissue	Histopathological alterations	Control	BA	HFCS	HFCS + BA
Histopatholology	Uterus	İnflammatory cell infiltrations	0.00 ± 0.00^c^	0.00 ± 0.00^c^	2.00 ± 0.75^a^	0.87 ± 0.35^b^
	Placenta	Hemorrhage	0.12 ± 0.12^c^	0.00 ± 0.00^c^	1.87 ± 0.83^a^	0.87 ± 0.35^b^
	Brain	Hyperemia and Hemorrhage	0.00 ± 0.00^b^	0.00 ± 0.00^b^	1.00 ± 0.75^a^	0.25 ± 0.16^b^
	Liver		0.12 ± 0.12^bc^	0.00 ± 0.00^c^	1.37 ± 0.51^a^	0.50 ± 0.18^b^
	Kidney		0.00 ± 0.00^c^	0.00 ± 0.00^c^	1.25 ± 0.46^a^	0.37 ± 0.18^b^
	Lung		0.00 ± 0.00^b^	0.00 ± 0.00^b^	1.37 ± 0.51^a^	0.25 ± 0.16^b^
Immunohistochemistry	Uterus	Caspase-3 expression activity	3.12 ± 1.55^c^	2.25 ± 1.48^c^	72.12 ± 4.32^a^	31.12 ± 1.55^b^
	Placenta		5.12 ± 2.16^c^	4.25 ± 2.76^c^	60.62 ± 2.13^a^	19.62 ± 2.13^b^
	Brain		2.75 ± 1.16^c^	1.62 ± 1.06^c^	20.75 ± 1.48^a^	10.00 ± 2.00^b^
	Liver		7.87 ± 2.69^c^	6.25 ± 2.49^c^	56.00 ± 5.15^a^	22.12 ± 4.67^b^
	Kidney		6.50 ± 2.67^c^	5.12 ± 2.64^c^	42.25 ± 6.84^a^	11.62 ± 2.06^b^
	Lung		5.12 ± 1.80^c^	4.50 ± 2.39^c^	55.37 ± 1.92^a^	18.12 ± 3.68^b^

^a, b,c^ Different superscripts in the same row showed statistically significant differences (*p* < 0.001). The same superscripts a, b, and c do not show statistically significant differences within the same row (*p* > 0.05). Data are expressed as mean ± standard error mean (SEM). One-way ANOVA followed by post hoc Duncan test was performed. BA: Boric acid; HFCS: High Fructose Corn Syrup

## Discussion

Boron is an important trace element of great importance for human and animal biology. Evidence from previous studies, both in vivo and clinical, indicates that boron makes a promising trace element. These studies demonstrated that boron-deficient rats [27] and frogs [28] had impaired reproductive performance and embryonic development. In our study, pregnant rats were given oral BA and HFCS for 18 days, and the changes in MDA and GSH levels in whole blood and SOD and CAT enzyme levels in erythrocyte pellets were investigated. Additionally, maternal uterus and placenta tissue, fetal brain, liver, kidney, and lung tissue were evaluated histopathological and immunohistochemically. TNF-α, IL-6, and leptin levels in the plasma were also measured. In our recent study, we reported that ascorbic acid prevents cadmium toxicity in maternal and fetal tissues by regulating oxidative imbalance and alleviating histopathological changes in pregnant rat tissues [29]. In this study, we investigated how oral administration of BA (20 mg/kg) during pregnancy affects the harmful effect of HFCS on maternal and fetal tissues.

All groups in this study received standard rat feed. Additionally, we added only tap water to the drinking water of the control and BA groups, along with rat feed, while we added F-30 solution to the drinking water of the HFCS groups daily. Interestingly, pregnant rats fed HFCS significantly reduced their food intake, a pattern similar to previous studies on HFCS-fed rats [30] and mice [31]. In our study, body weight increased periodically in the HFCS group due to pregnancy and the effect of HFCS. This finding was not consistent with the report that body weight level increased only numerically in rats fed an HFCS-supplemented diet [32]. This may be due to the study’s use of different doses of HFCS or the number of fetuses due to pregnancy. Interestingly, body weight did not change in the BA group despite pregnancy. This is in agreement with reports that BA reduces body weight in other pregnant [33] and non-pregnant [34] rats. In addition, HCFS administration did not statistically alter plasma leptin levels, whereas BA supplementation decreased them. Indeed, this situation is in agreement with the report by Toop et al. [35], which showed no difference in plasma leptin levels in pregnant rats fed with HFCS, and the report by Kucukkurt et al. [19], which demonstrated that boron supplementation decreased leptin levels. In contrast, the present study did not agree with Yazici et al. [36], who discovered that giving rats BA prevented the drop-in leptin levels caused by both exercise and ovariectomy. The period of boric acid administration, the dosage, or physiological variances among different animals may account for discrepancies across reports.

Maternal nutrition plays an important role in the fetus’s physiology from conception and throughout pregnancy, as it can influence the number and differentiation of cells in the blastocyst [37]. In our study, HFCS increased oxidative stress and organ damage in pregnant rats, whereas BA decreased oxidative imbalance and histopathological and immunohistochemical abnormalities. In recent years, there has been increasing scientific debate about the potential health effects of HFCS on endocrine and metabolic disorders [38, 39]. Additionally, there are many studies showings that BA can prevent or reduce the increase in MDA levels, an indicator of lipid peroxidation [14, 40]. In the present study, we found that HFCS treatment during pregnancy increased MDA levels, an indicator of lipid peroxidation, but decreased GSH levels and CAT enzyme activity. Similarly, Unsal et al. [30] showed that HFCS application caused oxidative damage by decreasing GSH levels as well as increasing MDA levels. The study’s findings align with previous studies, demonstrating that high fructose consumption triggers oxidative stress and lipid peroxidation by increasing MDA levels and decreasing SOD and CAT activities [41, 42]. The findings obtained in the study are similar to those in which BA supplementation increased antioxidant defense system levels by increasing SOD and CAT activity and significantly decreasing MDA levels [43]. Indeed, this demonstrated that HFCS significantly reduced the antioxidant capacity of pregnant rats, leading to excessive free radical production in the body, a condition that BA supplementation could prevent.

Any stimulation that occurred during the embryonic stage has the potential to trigger a fetal reaction and adaptation, which could result in long-term or irreversible abnormalities to the body’s structure or function [44, 45]. In the current study, we found that HFCS increased the levels of TNF-α and IL-6, but BA decreased such levels. This evidence suggests that HFCS-associated feeding may cause inflammation in pregnant rats, resulting in embryonic and fetal organ damage. This supports the findings of a prior study, which found that rats fed HFCS had considerably higher serum TNF-α and IL-6 levels [46]. Indeed, this is also consistent with the findings of Ekici et al. [47], who reported that HFCS increased TNF-α levels in muscle tissue. However, it was reported that IL-6 cytokine levels did not change at a statistically significant level in rats fed 21% high fructose diet compared to the control group [48]. These differences could be related to pregnancy, animal breeds, duration of administration, and administration doses.

It is generally accepted that increased oxidative stress or suppression of antioxidant systems due to a variety of reasons causes inflammation in the organism and may result in tissue damage [47]. Tepebaşı et al. demonstrated that the administration of HFCS to rats led to a significant increase in caspase-3 levels during the apoptotic process, accompanied by pronounced hyperemia, severe inflammatory cell infiltration in renal tissues, and vacuolar degeneration in tubular epithelial cells [49]. Youssef [50] also showed that high fructose consumption increased inflammatory cells in liver tissue, enlarged sinusoid regions, and significantly elevated caspase-3 levels. Similarly, our findings revealed that HFCS consumption caused histopathological damage and increased caspase-3 levels in fetal and maternal tissues. BA treatment reduced these pathological findings and caspase-3 immunohistochemistry levels in pregnant rats exposed to HFCS consumption. This finding supports the previous report showing that BA supplementation in rats with cyclophosphamide-induced liver toxicity was successful against immunohistochemical caspase-3 activity and histopathological changes in the liver [51].

Our findings clearly demonstrate that BA reduces the damage caused by HFCS on histopathology and immunohistochemistry, as well as oxidative stress and higher levels of pro-inflammatory cytokines and leptin. Consequently, BA (20 mg/kg) may have an ameliorative effect on maternal and fetal tissue damage and biochemical parameters caused by HFCS.

## Conclusions

This study demonstrated that exposure to HFCS induced an oxidative imbalance in pregnant rats, resulting in oxidative stress, inflammation, and pathological damage to the uterus, placenta, and fetal tissues. We found that oral administration of BA during pregnancy in HFCS-exposed rats reduced oxidative stress and inflammation and ameliorated damage to uterine, placental, and fetal tissues. Our findings support that oral BA supplementation may have a protective effect against HFCS-induced oxidative stress, inflammation, and tissue damage in the uterus, placenta, and fetuses of pregnant rats. In light of these findings, it is recommended that food manufacturers and processors prohibit the use of additives containing HFCS and implement warning labels on products containing HFCS. Furthermore, it is recommended to avoid high-calorie beverages containing HFCS during pregnancy and throughout life to safeguard the health of both the mother and her offspring. This will also provide valuable insight into the health effects of HFCS and BA during pregnancy, thereby guiding researchers in the design of future studies.

## Electronic Supplementary Material

Below is the link to the electronic supplementary material.

Supplementary Material 2

Supplementary Material 3

## Data Availability

The data that support this study will be shared upon reasonable request to the corresponding author.
