# Supplementary material for: The Effect of Boric Acid on Oxidative Stress, Inflammation, and Apoptosis in Embryonic and Fetal Tissues Damage Caused by Consumption of High-Fructose Corn Syrup in Pregnant Rats
Source: Reprod Sci. 2025 Jan 16;32(2):514–25. doi: 10.1007/s43032-025-01792-z (PMC11825574; doi:10.1007/s43032-025-01792-z)
Supplement: Supplementary file 1 — Supplementary Material 2 [file 43032_2025_1792_MOESM1_ESM.pdf]

T.C.
PAMUKKALE ÜNİVERSİTESİ
Hayvan Deneyleri Etik Kurulu

Sayı : E-60758568-020-371081 29.05.2023
Konu : Başvurunuz Hk.

Sayın Öğr. Gör. Mehmet BAŞEĞMEZ

İlgi : 16.05.2023 tarihli dilekçeniz.

212.253.207.178
3168
29.05.2023

"Yüksek Fruktozlu Mısır Şurubu ile Beslenen Gebe Sıçanlarda Borun Embriyonal Doku Gelişimi ve Trofoblast Hücre Proliferasyonu Üzerine Etkisi" (PAUHDEK-2023/16) konulu çalışmanız **24.05.2023 tarih ve 2023/04 sayılı** toplantımızda görüşülmüş olup,

Yapılan görüşmelerden sonra, söz konusu çalışmanın **Hayvan Deneyleri Etiği açısından uygun olduğuna** ve **28 adet (Wistar Albino) dişi sıçan kullanılarak yapılmasına** oy birliği ile karar verildi.

Gereğini bilgilerinize rica ederim.

Doç. Dr. Habip ATALAY
Kurul Başkanı

Bu belge, güvenli elektronik imza ile imzalanmıştır.
Belge Doğrulama Kodu :BSCA2RHLCR Pin Kodu :67782 Belge Takip Adresi : https://www.turkiye.gov.tr/pau-ebys

Adres:Tıp Fakültesi Dekanlığı Kınıklı/Denizli
Telefon:0 (025) 8 Faks:0 (258) 296 17 65
e-Posta:hadek@pau.edu.tr Elektronik Ağ:http://www.pau.edu.tr
Kep Adresi: paurektorluk@hs01.kep.tr

Bilgi için: Selda ERKİŞİ
Unvanı: Bilgisayar İşletmeni

Bu belge,güvenli elektronik imza ile imzanlamıştır.
