# Supplementary material for: The Effect of Boric Acid on Oxidative Stress, Inflammation, and Apoptosis in Embryonic and Fetal Tissues Damage Caused by Consumption of High-Fructose Corn Syrup in Pregnant Rats
Source: Reprod Sci. 2025 Jan 16;32(2):514–25. doi: 10.1007/s43032-025-01792-z (PMC11825574; doi:10.1007/s43032-025-01792-z)
Supplement: Supplementary file 2 — Supplementary Material 3 [file 43032_2025_1792_MOESM2_ESM.pdf]

T.C.
PAMUKKALE UNIVERSITY
Animal Experiments Ethics Committee

Number : E-60758568-020-371081 29.05.2023

Lecturer Dr. Mehmet BAŞEĞMEZ

Your study on **'The Effect of Boron on Embryonal Tissue Development and Trophoblast Cell Proliferation in Pregnant Rats Fed with High Fructose Corn Syrup' (PAUHDEK-2023/16)** was discussed at our meeting dated 24.05.2023 and numbered 2023/04,
And after the discussions, it was unanimously decided that the study in question was appropriate in terms of Animal Experiments Ethics and that it was carried out using 28 (Wistar Albino) female rats.

For your information.

Assoc. Prof. Dr. Habip ATALAY
Chairman of the Board

This document has been signed with secure electronic signature.

Verification Code :BSCA2RHLCR Pin Code :67782 URL: https://www.turkiye.gov.tr/pau-ebys
Faculty of Medicine Kınıklı/Denizli
Phone:0 (025) 8 Fax: +90 (258) 296 17 65
e-mail: hadek@pau.edu.tr URL:http://www.pau.edu.tr Kep e-mail: paurektorluk@hs01.**kep**.tr
For information: Selda ERKİŞİ

This document has been signed with a secure electronic signature.
